# Digital Health Interventions for People With Type 2 Diabetes to Develop Self-Care Expertise, Adapt to Identity Changes, and Influence Other’s Perception: Qualitative Study

**DOI:** 10.2196/21328

**Published:** 2020-12-21

**Authors:** Sophie Turnbull, Patricia J Lucas, Alastair D Hay, Christie Cabral

**Affiliations:** 1 Centre for Academic Primary Care Population Health Sciences Bristol Medical School, University of Bristol Bristol United Kingdom; 2 School for Policy Studies University of Bristol Bristol United Kingdom

**Keywords:** social identity, diabetes mellitus, internet-based intervention, health care disparities, socioeconomic factors, self care

## Abstract

**Background:**

A diagnosis of type 2 diabetes (T2D) results in widespread changes to a person’s life and can be experienced as an assault on their sense of self. The resources available to an individual influence how the individual adapts to their diabetic identity and subsequently engages in self-care. Digital interventions can be viewed as a resource that people can draw on to adapt to the diagnosis. However, there is an indication that people from disadvantaged groups find digital health technologies more challenging to access and use, which may increase health inequalities.

**Objective:**

This study aims to gain insights into how and why people with T2D use digital self-care technology and how experiences vary between individuals and social groups.

**Methods:**

A purposive sample of people who had used a digital intervention to help them self-care for their T2D were recruited for the study. Semistructured interviews were conducted, and data were analyzed thematically.

**Results:**

A diverse sample of 21 participants were interviewed. Participants used digital interventions to help them to understand and feel more in control of their bodies. Digital interventions were used by participants to project their chosen identity to others. Participants selected technology that allowed them to confirm and enact their preferred positive identities, both by avoiding stigma and by becoming experts in their disease or treatment. Participants preferred using digital interventions that helped them conceal their diabetes, including by buying discrete blood glucose monitors. Some participants used technology to increase their sense of power in their interaction with clinicians, whereas others used technology to demonstrate their *good*ness.

**Conclusions:**

The technology that people with T2D have access to shapes the way they are able to understand and control their bodies and support preferred social identities.

## Introduction

Type 2 diabetes (T2D) is a chronic disease that affects a large number of people and creates a significant burden for patients and the health services that support them [1-5]. Worldwide, there is a prevalence rate of 6059 per 100,000 people, and the prevalence continues to rise [6]. In the United Kingdom, 1 in 10 people over the age of 40 years now has T2D [6]. Self-care is the dominant medical paradigm for managing chronic illnesses in wealthy nations, such as the United Kingdom [7,8]. The self-care model holds the individual responsible for managing their chronic illness [9]. For example, in the case of T2D, people are asked to change their diet; lose weight; administer their own medications; and in some cases, monitor their own blood glucose levels [5,10]. The self-care model presumes that an individual has both power and accountability to change the factors that affect their health and expects the person to take control of their condition [11,12]. The focus of individual responsibility in the self-care model neglects the real constraints on people’s lives and the impact of social and economic circumstances on people’s choices [12-13].

The self-care model demands that people consider themselves to be ill and need to change as a result of their illness. Greater engagement with management of diabetes has been shown to be associated with incorporating disease diagnosis into one’s social identity [13,14]. It is argued that becoming chronically ill and the associated changes to lifestyle are a challenge to self-identity [14,15]. A new social identity must be negotiated and incorporated into their existing social identities [16]. If successful, the illness is experienced as *just a part* of them [16,17]. For those who are unable to reconcile the illness identity with other social identities, the diagnosis may present a sense of the *diminished self* [17,18]. Understanding how people incorporate illness identities is, therefore, crucial to understanding self-care.

However, another challenge of the self-care model is that it assumes that everyone has the resources and capacity to self-manage [19-21] and that managing oneself is desirable. In terms of the former, there is established literature demonstrating that people in more privileged social positions have greater access to resources that can be used to avoid risk and to adopt protective strategies [15,19-24]. Health literacy is a prime example of this; where it is built on a foundation of early advantage or disadvantage and mediates any benefit from health education [25]. Furthermore, the promotion of self-control as a desirable expression of agency and power is culturally specific [18]. This Eurocentric interpretation sees loss of control as indicative of chaos and moral failing [11,13,15,18]. Within this individualistic framing, diagnosis is understood as a loss of control. and the inability to manage the disease as an individual’s moral failure [15]. Those who struggle to manage their illness are stigmatized and may receive less support from professionals, friends, family, and other people with diabetes [11,15]. This means that some groups are (likely) served poorly by traditional education campaigns that promote self-care: those with fewer financial and social resources to enable change, those with limited health literacy, those who struggle to assimilate their illness into their social identity, and those for whom self-control is not an inevitable and moral good.

Digital health interventions are an increasingly common tool used for self-care for people living with T2D [26]. As Lupton [27] puts it “digital health technologies are positioned as contributing to lay people effectively becoming the ‘managers’ of their own health and health care.” One argument for this shift is that digital health may reduce inequalities by increasing access to health interventions and by providing interventions that are tailored to the needs of the user [28-30]. The literature on how digital technology may be useful in supporting identity shifts is beginning to build [31], and one study that comments on the identity work that happens in online diabetes support groups [32]. This qualitative interview study was conducted to explore the experiences of adults with T2D using digital self-care technology. Our aim is to explore how adult users talk about their use of digital interventions for self-management of T2D by examining how they spoke about their identity in relation to their technology use and their illness.

## Methods

### Overview

The methodological orientation used in this study was an inductive approach drawing on the aspects of grounded theory [33,34]. Data were gathered using semistructured one-to-one interviews.

Ethical approval was granted by the University of Bristol, Faculty of Health Sciences Research Ethics Committee (the approval letter is provided in Multimedia Appendix 1).

### Participants

Participants were approached face to face and by email through community and diabetes groups, with a focus on groups that served lower-income neighborhoods and Black, Asian, and Minority Ethnic groups. Adverts were also placed in the *Diabetes UK* web-based and print magazines. We sought out adults who had been diagnosed with T2D and had used a digital intervention to support them to manage their condition at least once and who spoke and understood English. Recruitment materials sought out a range of experiences with digital interventions, including those who did not like them. The recruitment materials asked anyone interested the study to complete a screening questionnaire (Multimedia Appendix 2) that enabled purposive sampling of participants to capture a range of experiences across different social groups. A total of 27 potential participants completed the screening questionnaire, and interviews continued until data saturation was reached for the major themes.

### Procedure

Theory and previous research on the lived experience of T2D, self-care, and the digital divide were used to develop the topic guide. The topic guide was also developed iteratively, with revisions made to reflect themes emerging from the analysis. The 3 iterations of the topic guide are available in Multimedia Appendix 3.

Interviews were conducted in person or by telephone, according to the preferences of the participants. In-person interviews were conducted in Bristol and Leicester, and participants could choose to be interviewed in their homes or in a hospital-based diabetes unit. Written and verbal information about the research was provided, and the participants provided consent ahead of taking part in the interview. Interview duration ranged from 35 minutes to 2 hours and 13 minutes, and they were audio recorded and transcribed verbatim. All the interviews were conducted by 1 researcher (ST). ST took field notes during and immediately after the interviews.

### Analysis

Analysis began soon after data collection was started and was ongoing and iterative. Interviewing continued until data saturation was reached, and no new data arose in relation to the key themes. Encrypted audio recorders were used to record the interviews. The audio was transcribed verbatim and anonymized, and the script was checked for accuracy and imported into NVivo 12 (qualitative data analysis software; QSR International) for analysis. Both the audio and the transcripts were stored on the secure servers at the University of Bristol and in accordance with the Data Protection Act 2018. A thematic approach was used to analyze the data [35]. Theoretical frameworks and evidence from studies exploring the lived experience of chronic illness, self-care and health inequalities was used to identify some major themes before coding, and further themes emerged from the data. The theoretical frameworks and evidence included chronic illness as an assault on personal identities; stigma, self-determination, and control in chronic illness; the moral component of self-care; and the influence of the socioeconomic context on how people adapt to a chronic illness and their ability to self-care for their condition. The first 3 transcripts were independently coded by 3 authors (ST, CC, and PL). This team reviewed the coded transcripts in a meeting and developed a coding structure by consensus. Regular meetings were held to review subsequent coding, and the coding structure was adapted to accommodate new themes. The full coding tree is available in Multimedia Appendix 4. Participants were provided with a summary of the main findings after the analysis was complete.

### Research Team and Reflexivity

#### Personal Characteristics

This study was conducted as a component of ST’s PhD, during which she received formal and informal training in qualitative methods and was supervised by CC and PL, who are senior academics specializing in qualitative research. ST’s previous qualifications were a BSc degree in psychology and an MSc degree in neuropsychology, and most of her training and experience was in quantitative methods, which may have had a bearing on the conduct and the interpretation of the interviews. CC has a background in anthropology, and PL has a background in psychology; however, both work on applied health topics now, with a focus on inequalities in health.

#### Relationship With Participants

The study participants were unknown to the authors before the commencement of this study. The participants would have known that ST was a researcher at the University of Bristol. The participants who were interviewed in person would be aware that the interviewer (ST) was a White woman in her 30s, who appears to be relatively affluent, at a healthy weight, and with no visible disabilities. The participants were aware that the aim of the study is to explore the use of technology to support the self-management of T2D; however, they would not have known that the authors were exploring accessibility and identity. ST’s position while conducting the interviews was that digital interventions have the potential to be beneficial for people with chronic conditions and that there are likely to be differences in the way people access and use technology, particularly reflecting social and cultural differences.

## Results

### Sample Description

A total of 21 people were interviewed. One person initially expressed an interest in the study and then refused participation because they were uncomfortable with the university standard procedures for the storage of interview data. The brief sample overview is shown in Table 1 and the individual participant profile is shown in Multimedia Appendix 5. The sample achieved diversity in gender, household income, and neighborhood deprivation. Most participants were White British (White British, n=17; Asian or British Asian, n=3; other White background, n=1) and Christian, and the majority of our sample were older and well-educated individuals. Two-thirds of the participants had a university degree or equivalent, and none of the participants reported leaving school before the age of 16 years. There were 6 potential participants who were not interviewed because their characteristics were similar to the majority of the sample (White British, well educated, and from more affluent neighborhoods) and, therefore, did not increase the diversity of the purposive sample.

**Table 1:** Participant characteristics (N=21).

Participant characteristics		Male (n=11), n (%)	Female (n=10), n (%)
**Education**			
	Secondary school or equivalent (low education)	1 (9)	0 (0)
	Intermediate between secondary level and university (eg, NVQ3-5^a^, diploma, and apprenticeship; low education)	5 (46)	2 (20)
	University degree or equivalent (high education)	5 (46)	8 (80)
**Estimated household income in the last year (before tax and not including benefits), £ (US $)**			
	Lowest income: <16,000 (<21,332) or eligible for means tested benefits	3 (27)	0 (0)
	Low income: 16,000-24,999 (21,332-33,330)	1 (9)	4 (40)
	Mid income: 25,000-34,999 (33,331-46,662)	3 (27)	0 (0)
	High income: 35,000-44,999 (46,663-59,994)	0 (0)	2 (20)
	Highest income: >45,000 (>59,995)	2 (18)	2 (20)
	Prefer not to say	2 (18)	2 (20)
**Use of digital interventions**			
	Lighter (≤2 interventions)	7 (64)	5 (50)
	Heavier (>2 interventions)	4 (36)	5 (50)
**Home neighborhood deprivation^b^**			
	1 Most deprived	1 (9)	2 (20)
	2 Lower SES^c^	2 (18)	1 (10)
	3 Mid SES	3 (27)	1 (10)
	4 Higher SES	1 (9)	2 (20)
	5 Highest SES	4 (36)	3 (30)
	Not available	0 (0)	1 (10)
**Age (years)**			
	21-40	1 (9)	1 (10)
	41-60	4 (36)	5 (50)
	61-70	6 (55)	4 (40)
	71-80	2 (18)	1 (10)

^a^NVQ3-5: National Vocational Qualification levels 3 to 5.

^b^Indices of multiple deprivation score derived from the participant's home post code were used to determine the participant's neighbourhood deprivation within the United Kingdom, and the quintile is given.

^c^SES: socioeconomic status.

The sample was evenly divided between light and heavy users of digital health interventions. Most participants did not use interventions designed specifically for people with diabetes but rather used technology designed to support healthy living and social connectivity. Wearable fitness trackers were the most commonly used intervention (16 participants) and apps that tracked nutrition or fitness (11 participants). The diabetes-specific interventions were the blood glucose monitors (BGMs; Dario meter, Freestyle Libre, and Trueyou mini) used by 10 participants (5 supplied by health care practitioners [HCPs] and 5 purchased privately) and 3 different apps each used by 1 participant (Diabetes diary, IBG star app, and Habits, a South Asian–specific diabetes app). The median number of different interventions trialed by the participants was 2 (range 1-7); 12 participants were lighter users (≤2 intervention) and 9 were heavier users (>2 interventions) of digital technology.

### Findings

As our focus is on how participants interacted with digital health interventions, we focused on the ways in which they were used regardless of whether they were light or heavy users of this technology. Most participants (both heavier and lighter users) used fitness trackers. Users who had tried a greater number and range of different types of technology were heavier users, whereas the 3 users who used diabetes-specific self-care apps (excluding BGMs) were lighter users.

Participants used technology to help them understand their body and to feel like they had more control over their bodies and their diabetes. Digital interventions were used by participants to resist stigmatizing illness identities and to project a positive identity. Participants also used technology to increase their sense of power or status in their interaction with HCPs.

#### Understanding Their Bodies and Making the Invisible, Visible

Digital interventions were used as a tool to help participants understand their bodies, to develop their expertise in self-care, and to keep them engaged in the long-term management of their condition. Participants talked about using feedback from BGMs to monitor their bodies and establish how their “*body works, how it reacts*” [Participant number 33], turning a relatively hidden illness into something visible and tangible:

I put myself to test my own blood, finger prick testing...because you have nowhere to hide from that evidence.

Participant number 33, female, high education, heavier user

One woman described herself as an “*inveterate self-experimenter*” [Participant number 41, female, high education, heavier user], describing trial-and-error experiments, in which she tested whether specific diets recommended on the web worked for her, using the output from monitors as evidence.

Similarly, feedback from wearable fitness monitors was used by participants to see changes in their own fitness or behaviors:

The heart rate monitor, it’s really good because...you can get out of breath but your heart rate...can come down very quickly. You realise that your fitness levels are going up....I felt really excited by it; liberated actually.

Participant number 37, female, high education, heavier user

I wouldn’t be without my Fitbit, it drives me. It absolutely drives me, because I get panicky last week when I couldn’t blooming recharge the thing...I have to know what I’m doing.

Participant number 10, female, low education, lighter user

Wearable technology supported their self-care by providing motivation to be more active and positive feedback on their achievements:

The monitoring [with a Fitbit] gives me a reward. (...)an aid to help me celebrate my achievement.

Participant number 30, female, high education, heavier user

#### Feeling in Control

Through the use of digital interventions, participants felt like they had more control over their diabetes and felt more in control generally. Being “*more informed*” [Participant number 26, male, low ed] about diabetes in general and having personalized information created a feeling of greater agency to affect their diabetic bodies, behavior, and health care:

I think the Garmin and the, erm, er, it, it is good really...the more you start seeing what your body is doing, the more fascinating it becomes, and you feel more in control, really.“

Participant number 37, female, high education, heavier user

One woman spoke about how having web-based access to her medical records made her feel like she had more ownership over her health care, feeling like she was “*part of it, and it’s not something the doctor owns*,” which made her “*a bit more focused to try and get*" her blood glucose levels “*better, get more under control*” [Participant number 31, female, low education, light user].

Digital interventions helped participants to feel more in control of their diabetes in situations where they were out of their normal routine. This included when people were in environments where they could not control what happens, such as holidays, and when they had changed their management strategies:

I- mainly [use the Freestyle Libre] when I’m at most risk of going off, off, erm, the wagon. (...) if I have any work trips and I’m staying in a hotel, I’ll slap one on, because that way, as I say, it gives me more self-control(...) without those two things [blood glucose meter and Freestyle Libre], I wouldn’t be in control of my blood glucose. I would, I would be thinking, “Oh well, just one won’t hurt, will it? This is a special dinner, I’ll have pudding.”

Participant number 41, female, high education, heavier user

#### Projecting Positive Identities

Participants used digital interventions to project, enact, and confirm their preferred positive identities. Through their use of technology, participants presented themselves as someone who “*got things under control*” [Participant number 27, male, low education, light user]. Participants often described the use of digital technologies as being associated with being younger, fitter, in control, and more skilled or educated; with higher status; or with specialized knowledge:

It’s probably wrong to try and box people in, but I don’t think it’s any point trying to tell an 85-year-old about Fitbits.

Participant number 27, male, low education, light user

...we’ve always had Fitbits....from when they were first introduced into the UK...we’ve just upgraded it to a newer model...some mornings I’ll get up and...I’ll just wear my Apple watch for the day just for something different.

Participant number 34, male, low education, light user

Some described how the technology they used required a level of understanding that not everyone had. One man talked about how he could understand the information he got from the Freestyle Libre because he had done a “*mathematics Open University, er, foundation course*” and consequently could “*understand a bit about the statistics*" [Participant number 42, male, high education, heavier user]. A woman described how she would look at the data from her Freestyle Libre each morning because she was a “*data master(...) someone who likes data*” [Participant number 33, female, high education, heavier user]. These were reflections of personal attributes rather than demographic differences in this sample; younger participants [Participant numbers 23 and 31] were lighter users, whereas others recognized that older people could acquire the right skills and knowledge:

...[for] the older generation apps it’s not really a thing...unless you’re, silver surfers....they have to understand what the app is doing or what is an app, ‘cause a lot of people don’t understand what an app is.

Participant number 38, male, higher education, heavier user

#### Defending Against the Stigmatized Diabetic Identity

The stigma felt by participants in relation to their diabetes diagnosis was apparent in interviews. Many of the participants described experiencing stigma as a result of being diagnosed with diabetes. There was a sense that family, friends, and the media blamed them for getting diabetes because they were overweight or (they presumed) they had an unhealthy lifestyle:

I remember somebody saying to me, “Well, do you think it’s ‘cause you probably drank too much?”

Participant number 11, female, high education, heavier user

...people think you’re a druggy....I remember doing it [injecting insulin],... in a council office, I was doing consultancy work and my director at the time said, “You shouldn’t be dong that here...”

Participant number 38, male, high education, heavier user

Some talked about being given unsolicited advice on diet and exercise from people in their social circle and HCPs:

...all the publicity around type 2 is entirely negative, so people think A), that I must have brought it on myself, (...) people assume that you’re not exercising and they start lecturing you about that

Participant number 30, female, high education, heavier user

One participant talked about being stared at when she injected insulin in a public place where a man “*kept looking and looking and despite how I kind of turned or, you know, tried to move away*” [Participant number 31, female, low education, and light user].

In response to this stigma, some participants described not disclosing their diabetes diagnosis in fear of being labeled with the stigmatized diabetic identity. The 2 youngest (aged 29 and 31 years) participants who were both of British South Asian ethnicity emphasized the challenges of being diagnosed young and how that affected their wish to conceal their diagnosis because they are “*embarrassed or ashamed of it*” and because of the fear that people would “*judge you*” [Participant number 23, 31 years, male, light user]. One woman talked about not wanting to be identified as “*the diabetic lady*,” as she felt it was reductive and “*inhumane*” [Participant number 33, female, high education, heavier user]. A man spoke about people in the South Asian community concealing their diabetes diagnosis, for fear of “*their family members being tarnished*,” and emphasized that this was a problem particularly women in the community because it “*could be a barrier for her future kind of marriage proposal*" [Participant number 26, male, low education, light user]. These experiences of stigma matter particularly here because participants explicitly preferred digital health innovations, as they helped them conceal their diabetes. For example, the equipment used could aid concealment or risk exposure. One woman talked about the blood glucose testing kits supplied by the National Health Service (NHS) being “*bulky*,” and the small monitor she purchased herself allowed her to be more “*discrete*” conducting self-care activities [Participant number 40, female, low income, light user]. In contrast, a younger woman “*didn’t tell anyone*” about her diagnosis; however, her condition was exposed to family members because they “*noticed*” the blood glucose “*machine*,” and she talked about how she wanted future technology to be “*a bit more discreet*” [Participant number 24, female, low income, light user]. Men also mentioned wishing to conceal, or not “*advertise*” [Participant number 22 and light user], their diagnosis, describing going to the “*gents*” or somewhere “*private*” to inject insulin [Participant number 20, male, low education, light user]. One man had stated he was happy to “*tell everyone*” about his diagnosis, but he felt strongly that the behavior of injecting insulin was “*not normal*” [Participant number 28, male, low education, light user]. Although none of the men in this sample mentioned the use of digital technology in this regard.

A rejection of a *diabetic identity* could be seen in the views of those who did not see diabetes as a progressive illness and believed that it was possible to reverse or halt their diabetes. They had not surrendered to their *sick self* and felt that their illness was something that they were still able to master. Participants described hearing stories where “*people lost weight and their diabetes actually went*” [Participant number 22, male, low education, and light user], which motivated them to go to the gym or seek out technology that could help them in their efforts “*Trying to reverse*” their diabetes [Participant number 23, 29 years, male, high education, light user], where medically they were no longer considered to be diagnosed with the condition. However, this also resulted in conflicting identities. One man was positive about technology because it had worked to reverse a diabetic diagnosis; however, he also acknowledged that he would always *have* diabetes:

[using apps] seems, it seems to have worked...if I went to the doctors now, I would no longer be diagnosed as diabetic. But, erm, but, that I am diagnosed as diabetic, means that, you know, in a sense, once you’ve got it, you know, you’ve got it.

Participant number 29, male, high education, heavier user

#### Increasing Status With HCPs

One of the ways that digital technology was used by participants to defend against an illness identity was as a tool to gain power in their interaction with HCPs. Some participants talked about using technology to resist treatment prescribed by clinicians or to modify their treatment regime. For example, one man bought his own blood pressure monitor to avoid taking medication:

obviously the GP didn’t like me not taking any blood pressure tablets. Er, so I said, well, “Then, we’ll keep an eye on it.” “If it starts going up, I’ll take the damn things.”

Participant number 36, male, high education, heavier user

Others described how gaining knowledge of diabetes online enabled them to negotiate care, as they knew more about their condition and treatment choices:

you’re slightly informed, so they can’t just treat you as somebody who’s, you know, like a naughty boy.

Participant number 26, male, low education, light user

One woman had received additional interest from her HCP and had been spoken to by medical students because of her weight loss, which she attributed to her Fitbit. She was treated as an expert patient, and through this, she felt a positive and desirable illness identity:

I said to him [GP], I braved him (...)when he asked me to see these students (...) I said to him, “If I’m doing all this for you...” Of course it’s helping me, I’m enjoying telling people about my journey [losing weight with the Fitbit]. And it is successful so far...So don’t get rid of me yet.

Participant number 10, female, low education, low income, light user

In *braving* the general practitioner, she describes both the power imbalance, her redress of this by providing a role as the expert patient through describing her journey to his students, and a demand she now felt able to make of him (that he does not get rid of her).

Some participants described using the digital interventions to provide proof of their management activities to their HCP, to demonstrate their *good*ness, and to avoid chastisement:

My diabetes nurse here, she’s quite pleased with me. And she said (...) “Oh, I wish everybody would have one [Fitbit]”

Participant number 35, female, high education, light user

## Discussion

### Principal Findings

Participants in this study used technology to help them understand their body, develop their expertise in self-care, and keep them engaged in the management of their condition. Through the use of digital interventions, participants could better understand and control their bodies and their diabetes.

Digital interventions were used by participants to project their chosen identity to others. Participants selected technology that allowed them to confirm and enact their preferred positive identities, both by avoiding stigma and by becoming experts in their disease or treatment. Participants preferred using digital health innovations that helped them conceal their diabetes, including by buying discrete BGMs. Participants used technology to increase their power or status in their interaction with HCPs. Some participants used technology to resist treatments prescribed by clinicians, to modify their treatment regime, or to negotiate support received. Others used digital interventions to provide proof of their management activities to their HCP, to demonstrate their *goodness,* and to avoid chastisement.

### Strengths and Limitations

To the authors’ knowledge, this is the first study to explore why and how people choose technology to support the self-care of T2D (or any chronic illness). Complete audio data was recorded for all interviews except one telephone interview for which the first 10 minutes were lost due to equipment malfunction. In the 3 telephone interviews, children and partners were in the vicinity of the participant during the interview, which could have impacted the interview content. The participants were not asked to comment on the transcripts. Double coding of a subset of interviews by 2 members of the team and an ongoing discussion about the coding structure ensured that the coding scheme was robust. Multiple views of the data promote confidence in the credibility of the findings [36]. A diverse range of experiences and opposing arguments were identified and presented.

Some caution should be exercised in the transferability of the findings. Although we sought out participants from less-advantaged and more ethnically diverse neighborhoods, our sample was predominantly White and well educated. Being able to speak and understand English was used as a study entry criterion, in response to the challenges of conducting cross-language qualitative research [37] and because of the lack of availability of resources to contract an interpreting service. This may have created a barrier to study entry for some minority ethnic groups. Although not all participants were technophiles, the sample mostly included adults aged over 50 years with an interest in technology. This sample may reflect historically lower access to the internet among people from groups of lower socioeconomic status and those living in remote geographical regions [38,39].

### Interpretations in the Context of Existing Literature

Most pertinent to the findings of this study is the literature on illness identities, stigma, and control of their bodies and illness [15,18,40-43]. The diagnosis of a chronic condition such as T2D has been described as an assault on the identity [14,44]. Successful adaptation is dependent on acceptance of the change to identity and associated lifestyle shifts and is mitigated by available resources [13,14,41]. In this study, participants described using digital interventions as a resource to support them to make a largely invisible disease more tangible, to help them understand their changed body, and to help them engage in self-care activities. They were using technology in a way that has been described as *digitizing the body*, providing a different way of *knowing* and *controlling* the health status of a body when physical sensations are an insufficient guide [27,32]. Digital tools increased participants’ sense of control and agency, implicitly reinforcing the idea of individual responsibility for managing their illness.

The majority of participants used technology to express preferred identities and to resist giving the master status to the diabetic identity. Luttrell [45] and James [46] proposed that people tell stories that allow them to present their more desirable selves in challenging situations such as the diagnosis of a stigmatizing disease. This was reflected in this study, where participants used technology to present more desirable preferred identities, such as *data master*, rather than the stigmatized diabetic identity. Both in the interview and in their social environments, they were able to demonstrate status by sharing their superior knowledge of technology. They used this knowledge to gain power and status in their interactions with HCPs.

The experience of enacted and felt stigma following a chronic illness diagnosis has been well documented, and this study demonstrated that stigma influenced the technology participants selected [15,47]. According to the stigma theory by Goffman [47], stigma occurs with chronic illness when people behave in a way that deviates from expectations of what is *normal*. He proposed that people conceal their true identities to fit in in the world of *normals* [47]. As T2D is a relatively invisible illness, people with the condition can choose to disclose their condition, which might mean they get support but might experience stigma [17,48]. Alternatively, they can conceal the condition to avoid identifying or being identified with the stigmatized identity [17,48]. In this study, some participants were able to replace big, bulky NHS BGMs with small *discrete* alternatives, which meant they were able to choose to not be socially identified or defined by their condition [18]. Those with more money were better able to buy technology that allowed them to pass as *normal*. The desire to avoid a stigmatized diabetic identity may also explain the widespread use of fitness trackers and the limited use of diabetes-specific digital interventions.

### Implications for Research and Future Intervention Development

The projection of identities through technology may be a promising route for the future development of technology to support people self-care for their T2D. In this study, there was a clear story being told by the participants with T2D about how they used technology to express positive identities and selected interventions because they supported their preferred identities. Participants in the most part did not select diabetes-specific technology and when they did, they talked about the importance of it being discreet or framed its use in terms of expression of positive identity. Some interventions have addressed identity change by providing educational modules, for example, the *Drink Less* app, which was designed to tackle excess alcohol consumption [49], and the MoveDaily intervention, which tried to link the formation of health habits to identity [31]. However, very few interventions aimed at supporting self-care of chronic conditions consider how using the interventions assists or prevents people’s ability to enact positive identities, for example, by designing diabetes-specific technology (such as a BGMs) to be attractive or to appeal to other positive identities. Intervention design that highlights people’s preferred identities may be more likely to be used and, therefore, have more beneficial effects.

### Conclusions

This study has shown that people with T2D used technology to understand and control their body and support their preferred social identity. Digital health technology was used to support the expression of positive identities (a good patient, an expert, or in control) or to avoid a stigmatized identity (by resisting the diagnosis or hiding their condition).

## Appendix

Multimedia Appendix 1 Ethics favorable opinion letter.

Multimedia Appendix 2 Screening questionnaire.

Multimedia Appendix 3 Topic guide.

Multimedia Appendix 4 Coding tree.

Multimedia Appendix 5 Participant individual profiles.

## Abbreviations

BGM: blood glucose monitor
HCP: health care practitioner
NHS: National Health Service
NIHR: National Institute for Health Research
T2D: type 2 diabetes
SPCR: School for Primary Care Research
