# Supplementary material for: Digital Health Interventions for People With Type 2 Diabetes to Develop Self-Care Expertise, Adapt to Identity Changes, and Influence Other’s Perception: Qualitative Study
Source: J Med Internet Res. 2020 Dec 21;22(12):e21328. doi: 10.2196/21328 (PMC7781797; doi:10.2196/21328)
Supplement: Multimedia Appendix 1 [file jmir_v22i12e21328_app1.docx]

### Appendix 1

| Ethics favourable Opinion letter **** |
| --- |
