# Supplementary material for: Digital Health Interventions for People With Type 2 Diabetes to Develop Self-Care Expertise, Adapt to Identity Changes, and Influence Other’s Perception: Qualitative Study
Source: J Med Internet Res. 2020 Dec 21;22(12):e21328. doi: 10.2196/21328 (PMC7781797; doi:10.2196/21328)
Supplement: Multimedia Appendix 3 [file jmir_v22i12e21328_app3.docx]

### Appendix 3

### Topic guide

**Version 1.0 (original version)**

**Version 1.3 (final version)**
