# Supplementary material for: Digital Health Interventions for People With Type 2 Diabetes to Develop Self-Care Expertise, Adapt to Identity Changes, and Influence Other’s Perception: Qualitative Study
Source: J Med Internet Res. 2020 Dec 21;22(12):e21328. doi: 10.2196/21328 (PMC7781797; doi:10.2196/21328)
Supplement: Multimedia Appendix 4 [file jmir_v22i12e21328_app4.docx]

### Appendix 4

### Coding tree

| Self |
| --- |
| Age related |
| Challenges to self healthy image |
| Embraced new image diabetic |
| Positive reinforcement from others |
| Externalising diabetes or othering |
| Feeling different or alien |
| 'Good diabetic' |
| Failure if illness progresses |
| Low carber |
| Psychological issues around diagnosis |
| Stigma |
| Being overweight |
| Bothering others |
| Taking insulin near children |
| Cultural stigma |
| Different for type 1 then type 2 |
| Doing too well |
| Experienced stigma |
| Felt stigma |
| From health care professionals |
| Judgement from other diabetics |
| Judgement from others |
| Not normal |
| Not share diagnosis with many people |
| Passing concealling diabetes |
| Problematic media presentation of T2D |
| Reductionist labelling |
| Young to have it |
| Technology |
| Features they like |
| Features they like |
| Comparative information |
| Case studies |
| Dietary advice |
| Logging calories |
| Recipes |
| Shows content of foods |
| Educational programme |
| Exercise videos |
| Fun vs boring |
| Humour |
| Info in case of emergencies |
| Medication options |
| Monitoring |
| Blood glucose levels |
| Blood pressure |
| Heart rat monitoring |
| Weight |
| Sleep tracking |
| Styling |
| Visual presentation of info |
| Tracking activity |
| Active minutes |
| Logging different types of exercise |
| Waterproof |
| Practical things they like |
| Alerts and Alarms |
| Automatic updating |
| Charge life |
| Data easy to extract |
| email updates |
| Gadget to make life easier emails etc |
| Larger formats |
| Reliable |
| Simple to use |
| Support from developer |
| Synchronises across devices |
| Wear rather than clipping on and off pedometer |
| How do people use digital interventions |
| Adapted tech to suit needs |
| Used for |
| Blood Glucose monitoring |
| Exercise |
| Heart rate monitor |
| Management of meds |
| Nutrition |
| Preparation for events |
| Using before Diabetes Diag |
| Ideal tech would contain |
| Features |
| Activity tracker |
| Active minutes |
| Bike |
| Breathing |
| Connect to clinician |
| Contents of food |
| Identify cals in food from picture |
| Monitoring |
| Blood glucose monitor |
| Blood pressure |
| Calorie tracking |
| Heart rate monitor |
| Medicine logging or reminders |
| Sleep tracking |
| More discrete |
| Discrete |
| Motivational feedback |
| Feedback after the event |
| Food excercise comparison calories burn |
| Give guidance on what should do |
| Propt to do activity on regular basis |
| Visual representation of info |
| Not invasive no needles |
| Personalised |
| Connected Drs notes |
| Cultural and language tailoring |
| Feedback on how food effects blood sugar |
| Lipid profile |
| Provide correct medication |
| Provide feedback 247 |
| Reminders of check ups |
| Tell you if having Hypo |
| Track ketones |
| Track metrics like body fat |
| Recipe guide step by step |
| Social element |
| Specifically diabetes related |
| Practical considerations |
| Can be used on phone |
| Conveience lifestyle features like alarms and phone calls |
| Different mediums for communication |
| Entertaining |
| Little input from user |
| Long battery life |
| More advanced |
| Reliable accurate |
| Simple to use |
| Watch |
| Waterproof |
| Limitations of tech |
| Access |
| No longer supported |
| Only available on certain phones |
| Phone has no reception |
| Tech not utilised properly by NHS |
| What to do with the information |
| Not meaningful |
| Only says what have done |
| Cost |
| Expensive |
| In app or product purchases |
| Not funded by NHS |
| Cost or access |
| Felt not for them |
| Instructions were incorrect or confusing |
| Not aware of features |
| Needle |
| Practical limitations |
| Basic layout on phone |
| Battery life |
| Forgetting to log or update |
| Forgoe design features in smaller model |
| Lack of support from developers |
| Light wakes you up |
| Lots of memory |
| Lots of set up |
| Not easy to use |
| Lots of logging |
| Not waterproof |
| Only has store bought food or not own brand |
| Only records walking or running |
| passwords |
| Patch comes off |
| Slow to load |
| Strap broke |
| Texts and emails etc |
| Too much detail |
| Tech for tech sake |
| What digital interventions |
| Access or cost |
| Age |
| Assets personal confidence |
| Know what tech to use |
| Hard to know which ones work |
| Barriers to uptake |
| Age |
| Cost |
| Data hard to understand |
| info not accessible |
| Support to use tech |
| Which ones to pick |
| Networks |
| Family friends |
| Received as present |
| Forums |
| Involvement in research |
| Through involvement in support group |
| Trusted person |
| Work |
| nhs |
| Health professional |
| Not available all phone brands |
| Others using tech with or for them |
| Personal skills confidence |
| Researched it |
| Technophile or phobe |
| Early adopter |
| Lack of awareness of tech not on radar |
| Support to set up |
| Technophile |
| Aesthetics |
| Brand loyalty |
| Construction of gender |
| Differences in forum use |
| Cultural differences |
| Novelty |
| Using tech unconsciously |
| Why do people use digital interventions |
| Digital social platforms their cultural relevance and influence on self care |
| Bypass medical model cultural consensus |
| Critical |
| Engaged in forum |
| Disengage bad |
| Forum tribalism |
| Improve access to information |
| Lurkers |
| Opinions |
| Motivational feedback |
| Breaks down limiting beliefs |
| Goal celebrations |
| Goal setting and challenges |
| Immediate feedback |
| Physical or visual prompts |
| Passing |
| Bulky |
| More discrete |
| More discrete or neater |
| Perceptions about technology |
| Accuracy of measuring or reporting |
| Accuracy will improve |
| Believes is accurate |
| Data not logical |
| Doesnt measure blood sugar interstitial fluid |
| Only measures while wearing limitations |
| Wear of have on you at all the time doesn't record while charging |
| Only need an indication of how doing |
| Just need to establish trend |
| Provides part of the big picture |
| Queries accuracy |
| Aimed at type 1 |
| Comparison to physical courses support |
| Complimentary |
| Compulsory vs chosen |
| Physical better |
| Background on how diabetes works |
| Getting people to do activity in physical intervention |
| Information from other diabetics |
| Physical prompts |
| Tech better than physical |
| Can access when want |
| Could get tech before got on course covered elsewhere renegotiation of treatment |
| Culturally sensitive information |
| Different types of people |
| personalised vs wide guidelines |
| Short vs long |
| Tech better |
| Unhelpful guidelines |
| Credibility |
| Personal confidence |
| Trusted body |
| Research studies |
| Trust brand |
| Trusted person |
| Family or friend |
| With diabetes |
| User reviews |
| Distrust may be agenda |
| Distrust peoples opinions |
| Online reviews eg apple store |
| Not helpful for diabetes |
| Cant use for driving check for hypos |
| Can't use for driving |
| Not motivating |
| Only works for motivated people |
| Older people eg care homes |
| Things stabilised |
| After a while not saying anything new |
| Lose motivation over time |
| To know if trustworthy |
| Bank details or hacking details |
| Insidious being watched |
| What are they doing with info |
| Which tech to trust |
| SECD differences |
| Age difference |
| Course older people |
| Culturally sensitive or appropriate woven throughout |
| Mental health |
| Encourage health anxiety |
| Social sharing |
| Competitions and supportive friend on tech |
| Comparitive information not people know |
| Diffusion of knowledge about tech through social group |
| As professional in the group |
| To other diabetics |
| Physical courses or support meet people |
| Emotional support |
| Online friends or support |
| Peer support |
| Talking point relate to non diabetics |
| Sharing achievements online |
| Social status of tech |
| Novelty |
| Gone out of fashion |
| Wanted to know what the fuss was about |
| Status conferred by brand or latest thing |
| Why do they use it |
| Feel in control |
| Control over care |
| Control over interactions with HCPs |
| Control over interactions with healthcare service |
| Of information they receive |
| Feel in control of health or diabetes |
| Feels gives control and self control |
| Not intrusive |
| Feeling have to do what computer tells you |
| Out of usual routine |
| Back up |
| Only need initially to learn about foods |
| People without tech have less understanding about whats going on |
| Self efficacy |
| Self efficacy only good for what you can change |
| Tech works because they have diabetes under control |
| Interaction with HCP |
| Access to clinicians |
| Avoid staff or system interaction |
| Avoiding medication |
| Feel ownership over care |
| Negotiate care |
| No waiting times |
| Plug gap in support not provided by NHS |
| Proof of activity |
| Replace dr diagnosis and treatment of opinion not part of why |
| Talking point with clinicians |
| Motivation to get tech covered in access |
| Family and friends |
| Health circumstance changed |
| Personalised care |
| Can do at own pace extract to TS |
| Can't get away from the data |
| Everyone is different |
| People without tech have less understanding about what’s going on |
| Personal coach |
| Trial and error |
| Wide guidelines vs detail |
| Tangible evidence |
| Supports management of diabetes |
| Associates health or beh changes with tech |
| Feedback confirmed by experience |
| Improved motivation |
| Breaking down limiting beliefs about excercise is |
| Tech used as a tool |
| Tool |
| Tool to support change |
| Trial and error |
| Understand body better |
| Can't hide from evidence |
| Checking on track |
| Prevention of illness |
| Picking up on illness before a problem |
| Evidence or feedback on physiology |
| Feel like an athlete |
| Indication of fitness |
| Interest in data |
| Link to trends in blood glucose |
| Understand how body responds to food |
