# Supplementary material for: Digital Health Interventions for People With Type 2 Diabetes to Develop Self-Care Expertise, Adapt to Identity Changes, and Influence Other’s Perception: Qualitative Study
Source: J Med Internet Res. 2020 Dec 21;22(12):e21328. doi: 10.2196/21328 (PMC7781797; doi:10.2196/21328)
Supplement: Multimedia Appendix 5 [file jmir_v22i12e21328_app5.docx]

### Appendix 5

Table 2. Participant individual profiles

| ID | Gender | Age | Ethnicity | Religion | Highest level of education | Estimated household income last year(before tax and not including benefits) | IMD quintile |
| --- | --- | --- | --- | --- | --- | --- | --- |
| 10 | Female | 61 | White- British | No religion | Intermediate between secondary level and university (e.g. NVQ3-5, diploma, apprenticeship) | £16,000 to £24,999 | 1 |
| 11 | Female | 59 | White- British | Christian | University degree or equivalent | £35,000 to £44,999 | 5 |
| 20 | Male | 74 | White- British | Christian | Intermediate between secondary level and university (e.g. NVQ3-5, diploma, apprenticeship) | £25,000 to £34,999 | 2 |
| 22 | Male | 67 | White- British | Christian | Secondary school or equivalent | £25,000 to £34,999 | 5 |
| 23 | Male | 31 | Asian or Asian British-Indian | Muslim | University degree or equivalent | <£16,000 and/or eligible for means tested benefits | 3 |
| 24 | Female | 29 | Asian or Asian British-Indian | Sikh | University degree or equivalent | £16,000 to £24,999 | 2 |
| 26 | Male | 48 | Asian or Asian British-Indian | Hindu | Intermediate between secondary level and university (e.g. NVQ3-5, diploma, apprenticeship) | £16,000 to £24,999 | 2 |
| 27 | Male | 58 | White- British | Prefer not to say | Intermediate between secondary level and university (e.g. NVQ3-5, diploma, apprenticeship) | Prefer not to say | 5 |
| 28 | Male | 66 | White- British | Christian | Intermediate between secondary level and university (e.g. NVQ3-5, diploma, apprenticeship) | >£45,000 | 3 |
| 29 | Male | 64 | White- British | Christian | University degree or equivalent | >£45,000 | 5 |
| 30 | Female | 56 | White- British | No religion | University degree or equivalent | >£45,000 | NA |
| 31 | Female | 58 | White- British | Christian | Intermediate between secondary level and university (e.g. NVQ3-5, diploma, apprenticeship) | Prefer not to say | 5 |
| 33 | Female | 60 | White- British | Christian | University degree or equivalent | Prefer not to say | 5 |
| 34 | Male | 55 | White- British | No religion | Intermediate between secondary level and university (e.g. NVQ3-5, diploma, apprenticeship) | £25,000 to £34,999 | 1 |
| 35 | Female | 63 | Other-White European, with mixed racial ancestry | Christian | University degree or equivalent | £35,000 to £44,999 | 3 |
| 36 | Male | 57 | White- British | No religion | University degree or equivalent | <£16,000 and/or eligible for means tested benefits | 4 |
| 37 | Female | 68 | White- British | Christian | University degree or equivalent | £16,000 to £24,999 | 4 |
| 38 | Male | 65 | White- British | Christian | University degree or equivalent | <£16,000 and/or eligible for means tested benefits | 5 |
| 40 | Female | 72 | White- British | Christian | University degree or equivalent | £16,000 to £24,999 | 4 |
| 41 | Female | 50 | White- British | No religion | University degree or equivalent | >£45,000 | 1 |
| 42 | Male | 72 | White- British | Christian | University degree or equivalent | Prefer not to say | 3 |
